# Population allele frequencies of disease associated SNPs in India: a paradigm shift from HapMap

**DOI:** 10.1186/1755-8166-7-S1-P105

**Published:** 2014-01-21

**Authors:** Pankaj Mankad, Srisanth Balan, Saleem Mohammed

**Affiliations:** 1 Xcode Life Sciences, 6B, Eldorado, Nungambakkam High Road, Nungambakkam, Chennai, 600 034, India

## Background

One of the challenges facing us in India, translating recent discoveries of chronic disease associated SNPs into clinical domain for prevention, is the lack of knowledge about the frequency of the polymorphism in our population. Hence, using available HapMap data has become a norm. However, it is anticipated that population genomics in India could be different from the HapMap Caucasian population. Relative disease risk prediction based on relevant SNPs, both for personalized medicine and for population genetics, has little value without accurate information of population allele frequencies. We report allele frequency of 384 SNPs directly related to chronic disease risk and metabolic traits in the Indian population.

## Materials and methods

We report the allele in a random sample of 146 individuals and compare them with the data reported in HapMap Caucasian population (n = 112). Genotyping was performed using Illumina golden gate genotyping assay following DNA extraction from saliva. Allele frequencies were determined by direct gene count method.

## Results

GWAS studies confirmed 384 SNPs to be associated with disease risk (364) of Diabetes Type 1 and 2 (54 & 118 respectively), Coronary artery disease(71), myocardial infarction(9), cardiac failure(24), sudden cardiac arrest(38), atrial fibrillation(9), hypertension(18), obesity(10), metabolic syndrome(2) and stroke(11); or were associated with metabolic traits (20). The master table of their ‘rs’ id, chromosomes, location and association is presented. Of the 384 SNPs, 44 were not in H-W equilibrium and were omitted. HapMap data were not available for 13 SNPs. We are reporting their allele frequencyon the Indian population for the first time. Of the remaining 307 disease association SNPs, statistically significant difference (p<.05) from HapMap Caucasian population was observed in 53% of them (164 of 307) and the difference of >10% (considered major in population genetics) was found in 42% (130 of 307). Of the 20 metabolic association SNPs, 50% (10 of 20) had statistically significant difference and in all of them it was >10%.

## Conclusions

We are reporting the largest repository, documenting disease and related SNP and allele frequencies in Indian population. We have also highlighted clear differences with HapMap data and would caution against indiscriminate use of HapMap for bench-to-bedside application of genetic knowledge in our population.

